# Purely electronic mechanism of electrolyte gating of indium tin oxide thin films

**DOI:** 10.1038/srep31239

**Published:** 2016-08-10

**Authors:** X. Leng, A. T. Bollinger, I. Božović

**Affiliations:** 1 Brookhaven National Laboratory, Upton NY 11973, USA; 2 Applied Physics Department, Yale University, New Haven CT 06520, USA

## Abstract

Epitaxial indium tin oxide films have been grown on both LaAlO_3_ and yttria-stabilized zirconia substrates using RF magnetron sputtering. Electrolyte gating causes a large change in the film resistance that occurs immediately after the gate voltage is applied, and shows no hysteresis during the charging/discharging processes. When two devices are patterned next to one another and the first one gated through an electrolyte, the second one shows no changes in conductance, in contrast to what happens in materials (like tungsten oxide) susceptible to ionic electromigration and intercalation. These findings indicate that electrolyte gating in indium tin oxide triggers a pure electronic process (electron depletion or accumulation, depending on the polarity of the gate voltage), with no electrochemical reactions involved. Electron accumulation occurs in a very thin layer near the film surface, which becomes highly conductive. These results contribute to our understanding of the electrolyte gating mechanism in complex oxides and may be relevant for applications of electric double layer transistor devices.

The (electric) field effect transistors (FETs), with insulating oxides used as gate dielectrics, are the basis of most modern electronics. In electrolyte-gated transistors (EGTs), the role of gate dielectric is played by an electrolyte — an ionic liquid or a polymer mixed with some ionic salt. EGTs have been explored as candidates for some niche applications such as organic thin film transistors for flexible, printed digital circuits, rollable displays, and conformal bioelectronic sensors^1^. One advantage of EGTs is that they enable accumulation of much larger density of induced mobile charge carriers in the gated channel, up to 10^14^–10^15^ cm^−2^. This has attracted much attention lately as a unique method of doping various materials up to metallic carrier density. In condensed-matter physics, this has emerged as a convenient technique to study quantum phase transitions (QPTs) from insulators to metals, or to superconductors. A great advantage is that electric-field charging is free of disorder (which is inherent to the more common chemical doping). Moreover, doping can be varied over a broad range in one and the same sample and in arbitrarily fine steps, just by tuning the gate voltage. A superconductor-to-insulator transition was traversed and studied in this way in SrTiO_3_, La_2−x_Sr_x_CuO_4_, YBa_2_Cu_3_O_7−x_ and ZrNCl, providing precious new information on the basic physics of superconductivity in these compounds^2^,^3^,^4^,^5^,^6^,^7^. Moreover, EGT technique is applicable to compounds that are not amenable to chemical doping. This provides a new strategy in the quest to discover new superconducting materials, of which electrolyte-gated KTaO_3_ has been the first successful example^8^.

However, the exact mechanism by which the applied external electric field affects the gated material and modifies its properties in complex oxides and other strongly-correlated electron materials has been a matter of vivid debate. The mechanism turns out to depend critically on whether the material is permeable to ions from the electrolyte, or not, and obviously this depends not only on the chemical composition and the crystal structure of the channel material but also on the ion size and nature. If the material is not permeable, the device behaves as the electrical double-layer transistor. In this case, the doping mechanism is purely electronic, with accumulation or depletion of charge carriers in the channel near the electrolyte/channel interface, quite similar to that in FETs. The difference is that in EGTs there is a concomitant redistribution of the ions of the electrolyte at the electrolyte/gate and electrolyte/channel interfaces, forming the so-called Helmholtz double layers, within which the electric field can reach the 10^7^–10^8^V/cm scale. This is an order of magnitude higher than what any insulator can withstand, which in turns brings in much higher induced surface charge density, up to 10^14^ cm^−2^, and even higher.

In contrast, if the channel material is permeable to ions, they will not stop at the interface but diffuse in until saturation is reached, as in fuel cells. In this case, the channel material undergoes electrochemical (Faradaic) doping that can extend throughout the entire film thickness. Such devices are referred to as the electrochemical transistors (ECTs) and explored for various electronics and sensor applications^1^.

In the recent EGT-based research on complex oxides, originally it was supposed that the charging process is purely electronic, i.e., accumulation or depletion of electrons occurs near the surface of the gated material. The problem with this scenario is that it is at variance with the observation that, in most cases, the entire gated film turned metallic upon electrolyte gating, even if it was more than 1,000 Å thick^9^,^10^,^11^,^12^. However, because in metals the density of mobile charge carriers is very high (in the 10^22^ to 10^23^ cm^−3^ range), the screening length is typically just a few Angstroms. In this case, one indeed expects that the field-induced effects would be restricted to a very thin (<1 nm thick) surface layer.

For this reason, uniform charging of thick films was rather attributed to some electrochemical reaction, such as massive electromigration and intercalation of oxygen ions or vacancies, as indeed substantiated for VO_2_ and SrRuO_3_ by incisive experimentation^10^,^12^. An implicit assumption has been that predominantly electrochemical mechanisms may be widespread and perhaps ubiquitous in complex oxides. It is thus of interest to test this hypothesis and search for a counter-example: a complex oxide resilient to electrochemical reactions, and susceptible to purely electronic charging when gated using an ionic liquid or a polymer electrolyte.

To differentiate between the two competing scenarios, note that electrochemical charging mechanism has several telltale characteristics that can be used as litmus tests. First, chemical intercalation is generally a slow process; under the typical experimental conditions it takes many minutes or even hours to reach the saturation charge density. Second, the charging process tends to be hysteretic; to discharge the sample one must apply the opposite voltage (and wait). In some cases, the intercalation process is irreversible, and can even be destructive, causing film delamination or complete chemical decomposition. Yet another litmus test, that we have devised, is to fabricate lithographically several devices (‘pixels”) for 4-point contact transport measurements next to one another. Then we apply electrolyte gating to one of them, say Pixel 1, and measure simultaneously the transport in this pixel, as well as in the first-neighbor Pixel 2, the next-nearest neighbor Pixel 3, etc. Note that the Pixels 2 and 3 are not covered by the electrolyte and hence not exposed to the large electric field. The two clear-cut limiting behaviors that this measurement can show are either (i) the transport properties of Pixel 1 show large changes that scale with the gate-voltage *V*_*G*_, while Pixels 2 and 3 remain unaffected, or else (ii) Pixel 2 also shows concomitant (although probably smaller) changes that scale with those in Pixel 1 and with *V*_*G*_. In the first case, we infer that the doping is predominantly electronic. In the latter case, there must be a substantial ionic diffusion and electro-migration, which is not limited to just the gated Pixel 1 but spreads into the neighbor pixel(s) because of the (small) lateral probe current.

In this paper, we report to have identified a complex oxide, indium tin oxide (ITO), in which electrolyte gating causes very fast charging, on the scale of a couple seconds, shows no hysteresis, and is completely reversible, suggestive of a predominantly electronic mechanism. Moreover, the changes in resistivity are only observed in the device that is covered by the gated electrolyte, with no signs of ionic diffusion into the neighbor devices.

ITO has been extensively studied due to its optical transparency in the visible range coexistent with high conductivity (up to 10^4^ S/cm at room temperature)^13^,^14^,^15^,^16^,^17^,^18^. The carrier concentration of ITO is in the range of 10^20^ cm^−3^–10^21^ cm^−3^, low enough to be amenable to substantial changes upon electrolyte gating^15^. For the field-effect experiments, electrolyte or otherwise, one needs high quality thin films with atomically smooth surfaces. Among various methods that have been used to prepare ITO thin films, magnetron sputtering stands out as having been proven capable of producing high quality films^18^, so this is the technique that we have adopted for the present study. So far, only a few studies focused on ultrathin ITO films^16^,^17^. The main conclusion was that the structure and the physical properties of ITO thin films are strongly dependent on the deposition conditions, so we have explored various growth protocols and substrates.

## Results

### Synthesis of epitaxial ITO thin films

In the present study, ITO thin films were synthesized on both single-crystal LaAlO_3_ (LAO) and yttria-stabilized zirconia (YSZ) substrates using RF magnetron sputtering (see methods). Prior to electrolyte gating experiments, every film was characterized by atomic force microscopy (AFM), X-ray diffraction (XRD), and transport measurements.

**Figure 1:**
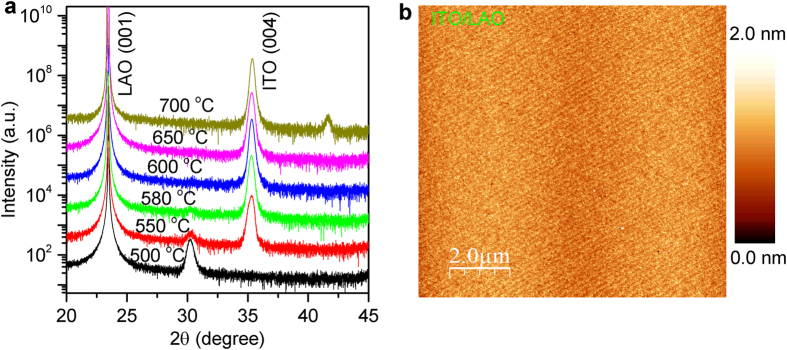
XRD patterns and AFM image of ITO films grown on LAO substrates. (**a**) Wide-angle (ω − 2θ) XRD patterns of ITO/LAO films grown at different temperatures as indicated. Except for the lowest trace, the curves are shifted upwards for clarity, but the relative scale remains the same. (**b**) AFM image of a 10 nm thick ITO film grown at *T*_*s*_ = 600 °C on LAO (001) surface. The rms surface roughness is 2.2 Å over the area of 100 μm^2^.

AFM and XRD data show that ITO films grown on either LAO or YSZ can have epitaxial alignment with respect to the substrate. Figure 1a shows XRD patterns for ITO films deposited at various growth temperatures on LAO substrates polished with the surface perpendicular to the crystallographic [001] direction. All these films were grown at the same radio-frequency (RF) power (*P* = 60 W) for the same time (*t* = 30 minutes), and in each case the measured thickness was about *d* = 30 nm. In the range 25° < 2*θ* < 40°, the XRD patterns of the films grown at *T*_*s*_ = 600 °C and *T*_*s*_ = 650 °C show a single peak (labeled as ITO 004 in Fig. 1), suggesting that the films are epitaxially alligned to the substrate. For films grown outside of this temperature window, i.e., at *T*_*s*_ = 550 °C or *T*_*s*_ = 700 °C, the XRD patterns show extra peaks. As the substrate temperature drops to *T*_*s*_ = 500 °C, only the ITO 222 peak appears in the XRD pattern, indicating that this film is also epitaxial but its orientation is different.

In Fig. 1b we present a typical AFM image of an ITO film grown on LAO substrate at *T*_*s*_ = 600 °C. One can clearly see steps and terraces and the average root mean square (rms) roughness of the surface is only 2.2 Å, indicating that the film surface is indeed atomically flat. However, the LAO substrate is generally heavily twinned, and this may affect the transport properties of ultrathin films grown on it^19^. Moreover, the lattice mismatch between LAO and ITO is substantial. In LAO *a*_*0*_ = 3.821 Å, while the lattice constant in ITO calculated from the XRD pattern is *a*_*0*_ = 10.156 Å; normalizing both to a reduced pseudo-cubic unit cell the calculated strain is +6.4% (tensile).

**Figure 2:**
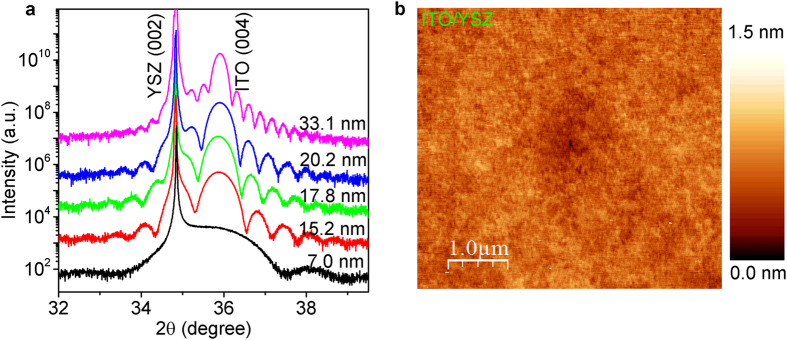
XRD patterns and AFM image of ITO films grown on YSZ substrates. (**a**) Wide-angle (ω − 2θ) XRD patterns of ITO/YSZ (001) films of different thicknesses as indicated. The curves are shifted upwards for clarity except for the lowest one, but the relative scale remains the same. (**b**) AFM image of a 10 nm thick ITO film grown at *T*_*s*_ = 600 °C on YSZ (001) surface. The rms surface roughness is 1.5 Å over the area of 100 μm^2^.

Thus, we speculated that YSZ may be a better choice of the substrate, because it does not twin, and since it has *a*_*0*_ = 5.12 Å it is much better lattice-matched to ITO, which would be under +1% tensile strain. Our experimental results confirmed this expectation. In Fig. 2a we show XRD patterns of ITO films grown on YSZ substrates polished with the surface perpendicular to the crystallographic [001] direction. These films were grown at *T*_*s*_ = 600 °C, with the remaining parameters the same as above, except that we also varied the film thickness. We can clearly see the so-called finite thickness (or Laüe) fringes, a clear sign that in a single-crystal film the surface and the substrate-film interface are perfect and parallel to each other on the atomic scale. In Fig. 2b we present the AFM image of a 10 nm thick ITO film on YSZ substrate. The rms surface roughness is only 1.5 Å, indicating that the film surface is atomically flat — as flat as the substrate itself.

### Electrolyte gating of ITO films

**Figure 3:**
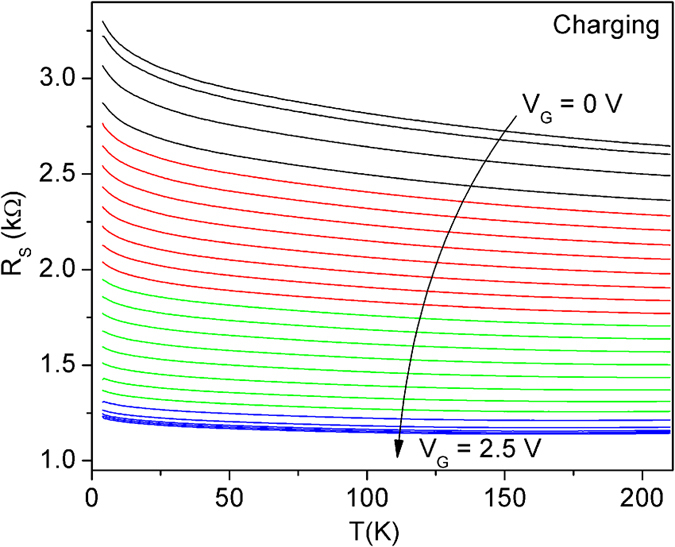
Transport results of an ionic liquid gated ITO film. Sheet resistance of a 10 nm thick ITO/YSZ film as a function of temperature as the gate voltage is increased from *V*_*G*_ = 0 (top) to *V*_*G*_ = 2.5 V (bottom). The gate voltage was varied in steps of *ΔV*_*G*_ = 0.1–0.2 V.

After the structural characterization, the ITO/YSZ films were patterned into a Hall bar configuration for transport measurements. In our gating experiments, we have used both an ionic liquid N,N-diethyl-N-methyl-N-(2-methoxyethyl)ammonium bis(trifluoromethylsulphonyl)imide (DEME-TFSI) and a polymer electrolyte (sodium fluoride salt dissolved in polyethylene glycol, PEG-NaF), and the results turned out to be quite similar. In Fig. 3 we show the dependence of the sheet resistance (*R*_*S*_) on the temperature, for various values of the *V*_*G*_. As the gate voltage increases from *V*_*G*_ = 0 to *V*_*G*_ = 2.5 V, the room temperature resistance drops by more than 50%, and then saturates. The sample remains insulating down to the lowest temperature. However, at higher temperatures (*T* > 120 K), the temperature coefficient of resistance changes from negative (semiconductor-like) to positive (metallic-like), as the gate voltage increases.

**Figure 4:**
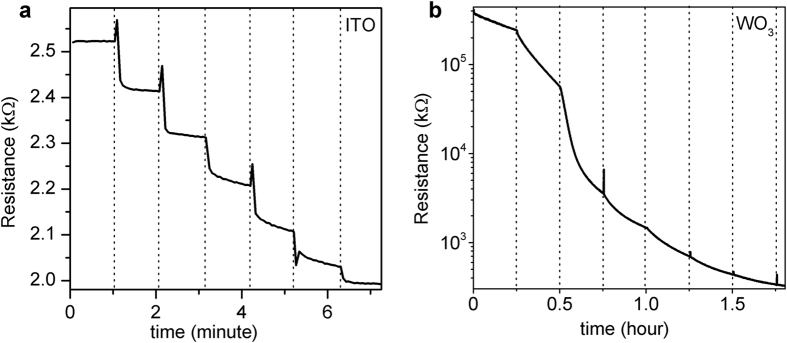
Resistance vs. time during charging for ITO and WO_3_ films. (**a**) As an ITO film is charged using DEME-TFSI as an electrolyte, the resistance drops immediately after we change the gate voltage, and then it saturates at each voltage. The dashed lines indicate the time when the gate voltage is changed and each voltage step is 0.1–0.2 V. (**b**) For WO_3_, with the same gate voltage step and the same ionic liquid, the resistance keeps dropping even after half an hour after we change the gate voltage, and it never saturates at each voltage step.

In the previous electrolyte gating experiments with various complex oxides and correlated electron materials, the usual charging time (needed to reach saturation at a fixed *V*_*G*_) was reported to be in the range of 10 to 30 minutes for each gate voltage step^2^,^3^,^4^,^5^,^6^,^7^,^8^,^9^,^10^,^11^,^12^. However, we have found that the charging time in ITO is extremely short. In Fig. 4a, we can see that for ITO the resistance drops immediately after we change the gate voltage, and then it saturates at each gate voltage. The charging time for each voltage step is about 1 minute, but most (>80%) of the resistance drop occurs within the first second or so. For comparison, in Fig. 4b we also show the results of gating WO_3_ films that were deposited in the same RF sputtering system^20^ and using the same ionic liquid electrolyte, DEME-TFSI. One can see that in WO_3_ (and other materials), the resistance keeps dropping even after half an hour after we change the gate voltage, and it never saturates at each voltage step. This striking difference indicates that the charging mechanisms in ITO and WO_3_ are most likely different. Since electrochemical reactions usually take a long time to proceed while electrostatic charging occurs essentially immediately, we infer the electrolyte gating in ITO is probably a purely electronic effect while in WO_3_ (and many other materials) electrochemical processes are involved.

**Figure 5:**
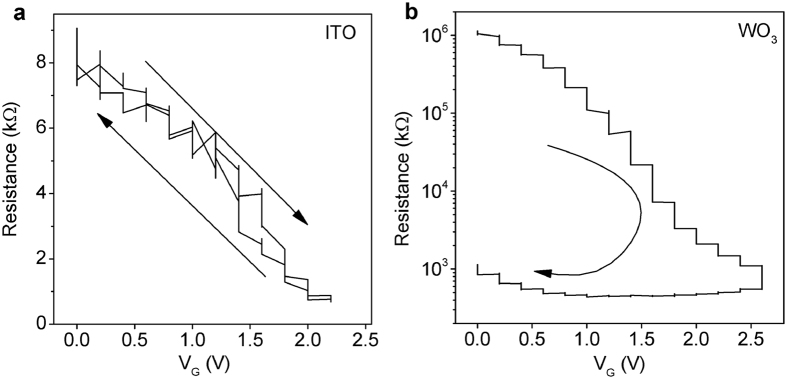
Resistance vs. gate voltage during charging and discharging for ITO and WO_3_. (**a**) For ITO, the charging/discharging time at each voltage step is less than one minute. The ITO film discharges completely and fast upon switching off the gate voltage. (**b**) For WO_3_, the charging/discharging time at each voltage step is over half-an-hour. A large hysteresis appears and one has to apply a negative gate voltage to fully discharge it.

In Fig. 5, we can see that the charging/discharging loop for ITO shows no hysteresis. Again, we contrast this with WO_3_, in which a large conductivity change persists after the gate voltage is removed. To restore the initial state in WO_3_ (before the positive gate voltage was applied), one has to apply the gate voltage of opposite (negative) polarity, and this process is also slow. Thus, this is another piece of evidence pointing to an electronic mechanism in ITO, in contrast to an electrochemical process in WO_3_.

**Figure 6:**
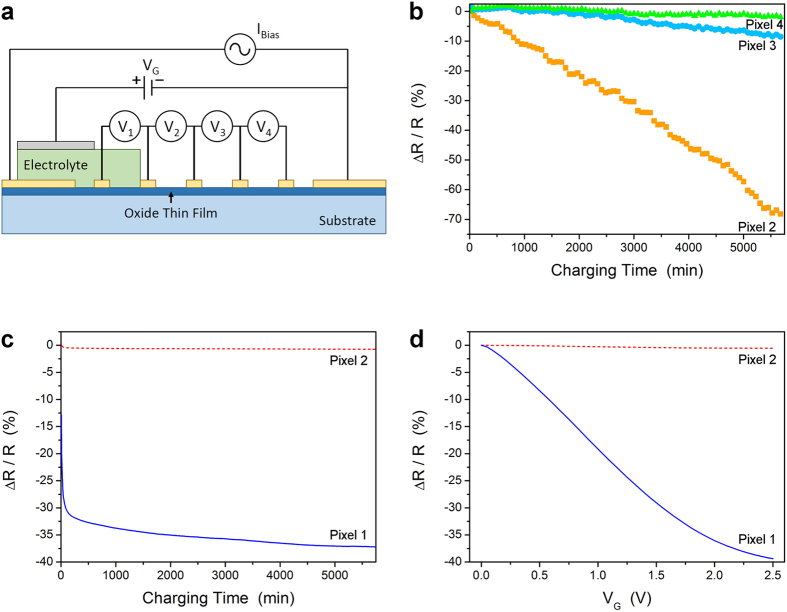
Influence of electrolyte gating outside of the charging region in ITO and WO_3_. (**a**) Cross sectional diagram of the Hall bar device geometry used in this work to test for long range effects in oxide thin films away from the area being charged. The bar was current biased and the voltage of the n^th^ pixel read by the voltmeter indicated by V_n_. The gate was a platinum mesh connected to a DC power supply. The spacing of voltage contacts was 300 μm for all devices. Several Hall bars for each material were tested with widths ranging from 10–200 μm. (**b**) The change in resistance for WO_3_ from the value observed at *V*_*G*_ = 0 V. The gate voltage was changed to +2.5 V in a stepwise manner at the beginning of the measurement. The resistance of Pixel 2 (■), the pixel closest to the electrolyte, changed dramatically over time. The resistances of Pixel 3 (●) and Pixel 4 (▲) also dropped but to a lesser extent with their increasing distance from the gated region. (**c**) In ITO the resistance of Pixel 2 (dashed line) hardly changes at all while the resistance of Pixel 1 (solid line) shows a large drop in a time scale orders of magnitude faster than the charging observed in WO_3_. (**d**) The change in resistance for Pixel 1 (solid line) and Pixel 2 (dashed line) again show large changes and essentially no changes, respectively.

In Fig. 6 we show the result of a new type of experiment that we have devised to conclusively test whether electrolyte gating causes massive ion intercalation into or removal from the gated material. We pattern the film into a long bar with multiple contact leads, as shown in Fig. 6a. This enables simultaneous measurements of the longitudinal resistance and/or the Hall effect in a number of segments of the bar (“pixels”). We have used this type of parallel transport measurement extensively in our experiments on films with combinatorial spread in the chemical composition^21^. The innovation here is that we pick one pixel (call it Pixel 1), cover it with electrolyte and gate it under varying conditions (temperature, gate voltage) while measuring the electrical transport simultaneously in that pixel and in the neighboring pixels (Pixel 2, Pixel 3, etc.) that are not covered by the electrolyte. In this case, only Pixel 1 could experience the extremely high electric field (up to 10^8^ V/cm) within the Helmholtz double layer. Given the small lateral probe current (typically 100 μA), the lateral electric field is 6–8 orders of magnitude smaller. Moreover, the electron screening length is 5 orders of magnitude shorter than the pixel length, and therefore any electron depletion or accumulation in Pixel 2 would be confined to a minuscule fraction of its volume. Altogether, if electrolyte gating causes only electronic processes, there could be no observable changes of the longitudinal or Hall resistance in Pixel 2, Pixel 3, etc. Conversely, if any such changes are detected, they could only come from electromigration of ions. If the ionic conductivity of the gated material is non-negligible under the actual experimental conditions (temperature, gate voltage), the (small) lateral probe current could drive some ions into the Pixels 2, 3, etc., which could produce a measurable change in electrical resistance.

In Fig. 6b we show that the later effect is in fact quite dramatic in thin films of WO_3_. Upon gating Pixel 1, as its resistance drops with time, there is a concomitant drop of resistance of Pixel 2 and Pixel 3 (although smaller for Pixel 3 by about a factor of 10), and the two track one another in time. Pixel 4, the furthest from the electrolyte-gated Pixel 1, shows the least change. The charging process in all of the pixels is slow, which is yet another indication of the electrochemical nature of the doping process. Figure 6c shows a complete absence of such an effect in ITO; Pixel 2 shows no changes whatsoever. The charging process is much faster, in particular the initial precipitous drop in Pixel 1 that occurs on a scale of seconds or less. Figure 6d also shows charging of Pixel 1 in ITO as a function of gate voltage, and the absence of any observable charging of Pixel 2. Altogether, these are incisive experiments that clearly demonstrate a dramatic difference in the charging mechanisms in WO_3_ and ITO; it is predominantly electrochemical (i.e., ionic) in WO_3_ and predominantly electronic in ITO.

## Discussion

If our inference is correct, and the observed modifications of the electrical resistance of ITO films originate from electron accumulation, then the affected layer must be very thin, just a few Angstroms thick. In this case, since the conductivity of the underlying layer remains the same, the change in the conductivity of the modified top layer must be much larger than the apparent change in the entire film. To make this more concrete, consider the data in Fig. 3 at some fixed temperature, e.g., *T* = 200 K. Upon electrolyte gating, the sheet resistance drops from *R*_*S*_ = 2.7 kΩ/□ to *R*_*S*_ = 1.15 kΩ/□, i.e., by about a factor of 3. If we assume that only the top layer about 5 Å thick is modified, while the rest of the film (95 Å thick layer) is unaffected, this would imply that the conductivity of the top layer has increased by a factor of about 30, to more than 2 × 10^5^ S/cm — comparable to those in good metals (say, nickel). While this is just a crude estimate, it ensures that the electron accumulation in the top layer must be massive, with the saturated electron density in the 10^23^ cm^−3^ range.

A question left open is why even at our highest gate voltage the resistivity slope changes sign at low temperature to negative (*ρ* increases as *T* is decreased). The two most likely explanations are as follows. First, the surface charge density induced by electrolyte gating may not be perfectly uniform, and hence one could imagine that (relatively large) metallic domains are embedded in a semiconducting matrix; as the temperature is decreased, these semiconducting barriers become more resistive and eventually dominate the resistivity of that surface layer. The other possibility is that rather than in real space, the ‘phase separation’ occurs in the k-space, e.g. by formation of a charge-density wave that partially gaps the Fermi surface along some azimuthal sectors that grow as the temperature is lowered. To decide between these scenarios one would need additional experiments that are outside the scope of the present study. Nevertheless, the fact that we can induce a highly metallic surface state by electric field provides an encouraging perspective for potential applications. Greatly increased charging/discharging speed (compared to WO_3_ and the like) is certainly another positive factor.

It is of interest to make a brief comparison with somewhat more broadly related experiments in the literature. One study similar in spirit to the present one was done on well-characterized, single-crystal SrTiO_3_ samples with atomically smooth surfaces^22^. Systematic transport measurements were made on lithographically defined devices with the Hall bar geometry. The four-point contact resistance and the Hall coefficient were measured, providing accurate values of the sheet conductance σ_*s*_, the sheet carrier density *n*_*s*_, and the Hall mobility *μ*. EGT experiments were made using a polymer electrolyte (polyethylene oxide with KClO_4_). (Dis)charging was done at *T* = 320 K, where the electrolyte was in a liquid state. The SrTiO_3_ sample is initially an insulator with a band gap of 3.15 eV. Electrolyte gating can turn its surface metallic, and a two-dimensional superconducting state emerges^23^ below the critical temperature *T*_*c*_ = 0.4 K. The doping mechanism apparently depends on the voltage bias. In the low-bias regimes, *V*_*G*_ < 3.7 V, the sample charging and discharging occurred within a second, and it is fully reversible. The maximum induced carrier density reaches *n*_*s*_ ≈ 10^14^ cm^−2^. This changes dramatically for *V*_*G*_ > 3.75 V. A leakage current, *I*_*G*_, shows a slow decay upon turning *V*_*G*_ on, and a negative value upon *V*_*G*_ removal. This high-bias process is irreversible; persistent conduction is observed even after removal of the gate bias. In this regime, the sheet carrier density can reach *n*_*s*_ ≈ 10^15^ cm^−2^. However, the thickness of the conductive region was deduced to be around 10 μm, which was supported by transmission electron microscopy (TEM). This was ascribed to some electrochemical process. The two candidate reactions are intercalation of K^+^ cations into SrTiO_3_ and oxygen removal. Secondary ion mass spectrometry (SIMS) showed no presence of K^+^ in SrTiO_3_, so it was concluded that the conductive layer is formed by oxygen removal from a few micrometers deep near-surface region of SrTiO_3_. Reflecting now on our present study focused on ITO, the main difference is that SrTiO_3_ is known to be prone to oxygen vacancy formation, and thus may be more analogous to VO_2_. This warrants some additional experimental study, but this is outside of the scope of the present paper.

In another interesting recent paper^24^, electrolyte gating of WO_3_ was studied by electro-chemical techniques. Experiments were largely complementary to ours, in several respects. First, there is a big difference in the film morphology. The WO_3_ films were grown by sol-gel technique at room temperature on conducting glass substrates and post-annealed in flowing oxygen at 400–550 °C for 30 min, and described as meso-porous^24^. From XRD patterns shown, they appear polycrystalline. While no morphological information has been provided in ref. 24, scanning electron microscope (SEM) images in an earlier report on similar films^25^ show disconnected grains, with the size typically on ~10 nm scale. Two ionic liquids were used, 1-methyl-1-butylpyrrolidinium bis-(trifluoro-methylsulfonyl)imide or [PYR_14_][TFSI] and 1-ethyl-3-methylimida-zolium bis(tri fluoromethylsulfonyl) imide or [EMIM][TFSI]. They have different ion sizes; the radius of [PYR_14_] is 3.9 Å, and for [EMIM] it is 3.5 Å. The key data shown are cyclic voltammetry, the dependence of source-drain current on *V_G_*, and X-ray diffraction. The main conclusion is that despite the large size of the ions, the doping mechanism in the films cannot be purely electrostatic since (a) the cyclic voltametry shows a clear Faradaic behavior, (b) the charging process is relatively slow (the tail in the source-drain current *I*_*sd*_ extends for several minutes), and (c) the doping charge estimated from the specific capacitance of the ionic liquid and the surface area of the WO_3_ exposed to the electrolyte is much smaller (only about 10%) than the measured charge. On the other hand, estimating the electrochemical doping charge needed to dope W from the oxidation state +6 to +5, the authors conclude that only 3% of the WO_3_ volume is doped if [EMIM][TFSI] is used as the electrolyte, and even less, 1.2% in the case of [PYR_14_][TFSI]. This is attributed to the large size of the cations of the ionic liquid that cannot be inserted in the relatively small cages of WO_3_. This led to speculation that a third mechanism may be at play as well for electrolytes made up of ions with relatively large size that cannot be inserted in the oxide lattice: nonconventional electrochemical doping, confined at the electrolyte/metal oxide interface. In summary, the authors proposed that doping of meso-porous WO_3_ films includes all three contributions — an electrostatic process, a conventional electrochemical process (protons from water traces are inserted into the WO_3_), and nonconventional interface-confined electrochemical doping, in which the injected electrons are compensated by the large cations of the ionic liquid packing at the interface.

Turning back to ITO, we note that there is a possibility of some electrochemical process, likely limited to the interface, and perhaps responsible for the tail in *I*_*sd*_ that extends for about a minute. (Alternatively, this could come from changes in the ionic liquid itself.) But we stress that most of the change occurs very fast and should be of electronic nature; this is the dominant effect in ITO.

In terms of the material investigated, the previous work most directly related to this one is a study of EGTs made of ITO films^15^. It is also complementary to the present work, in several respects. (a) The first major difference is in the film quality, structure, and morphology. ITO films studied there were amorphous, porous, and granular. Note that the material of which the grain boundaries are composed has a different (and unknown) structure and chemical composition, while it is precisely this material that is in direct contact with the ionic liquid. (b) They used a different ionic liquid, 1-ethyl-3-methylimidazolium bis(trifluoromethylsulfonyl)imide (EMI-Beti). (c) They performed two-terminal ac resistance measurements, using a frequency response analyzer to measure the dependence of the complex impedance on *V*_*G*_. Since both the capacitance and the resistance of the ionic liquid change with the gate voltage, it is conceivable that in these ac measurements the changes in properties of the ionic liquid play a role. (d) The doping mechanism was not discussed. However, since for positive *V*_*G*_, which causes electron accumulation in the ITO channel, equilibrium was reached within ~100 ms, we take this a signature of purely electronic process. (e) In contrast, for *V*_*G*_ < 0 (electron depletion) the time to reach equilibrium was orders-of-magnitude longer, ~100 s; this appears as an indication for some electrochemical process, but regrettably no further details were reported.

Comparing this with the present study, we conclude that there is a basic consistency, at least insofar as our basic conclusion about the doping mechanism being predominantly electronic in nature (for *V*_*G*_ > 0) is concerned. Our samples are high-quality, epitaxial, single-crystal, stoichiometric ITO films, and moreover, before the measurements of the temperature dependence of the resistivity we clean the surface by fast cycling. Thus, our results should be more characteristic of the intrinsic behavior of ITO crystals. Next, we have performed four-point-contact dc resistance measurements as a function of temperature down to *T* = 4 K, checking for possible superconductivity (none was observed down to this temperature), which of course was the primary motivation for the present as well as for almost all other recent studies of EGTs in complex oxides. Next, we have compared the behavior of ITO to that of single-crystal WO_3_ films under the same charging conditions, and showed that they have quite different intrinsic behavior. We have shown that there is substantial lateral ionic diffusion in WO_3_ while none is observed in ITO by a new type of experiment, where we lithographically pattern the film into a series of devices for 4-point contact resistivity measurements, expose one to electrolyte gating and monitor its resistivity simultaneously with that in neighboring devices. In the case of WO_3_, we have found that neighboring devices, even though they are not covered with electrolyte, show changes in resistivity that track those in the gated device, while no such effect is observed in ITO. Thus the charging mechanism is apparently material-dependent and different in these two cases, predominantly electrochemical in WO_3_ and predominantly electronic in ITO. Finally, we infer that in ITO the (electronic) charging must be restricted to a very thin layer near the film/electrolyte interface, implying that the local change in mobile carrier density is quite large, saturating at ~10^23^ cm^−3^.

Apart from the structure of the electrolyte itself, the crystal structure of the gated material plays a role and determines which doping mechanism will dominate upon electrolyte gating. In very open oxide structures with high propensity for oxygen vacancy formation, and high ionic mobility of oxygen, of which VO_2_ may be the best-studied example, the dominant mechanism can be oxygen electro-migration. WO_3_ is apparently less susceptible to this mechanism of ionic conduction because the vacant B site at the center of the perovskite unit cell is surrounded by six O^2−^ ions as nearest neighbors, so it readily accepts small cations, such as Li^+^. Indeed, H^+^ (i.e., a proton) is the smallest cation and, if present in sufficient concentration, dominates the ionic conductivity of WO_3_ (X. Liang *et al*., unpublished). Apparently, neither of these mechanisms is operative in ITO, by virtue of its ‘more compact’ structure.

In summary, we have grown high-quality epitaxial ITO films on both LAO and YSZ substrates by rf magnetron sputtering. Electrolyte gating triggers a large resistance drop under a small gate voltage. The process is very fast; a drop by over 80% occurs within a couple of seconds after the gate voltage is applied). No hysteresis is observed in the charging/discharging loop and the device next to the one that is electrolyte gated shows no changes in resistivity. Collectively, these three findings indicate that in ITO the primary effect of electrolyte doping is of electronic nature. This contrasts with previous studies of electrolyte gating of various complex oxides where the predominant effect is an electrochemical reaction.

## Methods

ITO thin films were synthesized on both LaAlO_3_ and yttria-stabilized zirconia substrates using RF magnetron sputtering. A sintered ceramic ITO (In_2_O_3_:SnO_2_ = 90:10 wt%) target was employed. The substrate temperature during film growth was varied from *T*_*s*_ = 500 °C to *T*_*s*_ = 700 °C. The chamber pressure during growth was *p* = 60 mTorr with an O_2_/Ar ratio 4:1. The growth rate was about 1 nm/minute. The film thickness was determined *ex-situ* using X-ray reflectivity measurements.

## Additional Information

**How to cite this article**: Leng, X. *et al*. Purely electronic mechanism of electrolyte gating of indium tin oxide thin films. *Sci. Rep.* **6**, 31239; doi: 10.1038/srep31239 (2016).
